# Magnetic Resonance Imaging Evaluation in Patients with Linear Morphea Treated with Methotrexate and High-Dose Corticosteroid

**DOI:** 10.1155/2018/8391218

**Published:** 2018-07-02

**Authors:** Mohammad Shahidi-Dadras, Fahimeh Abdollahimajd, Razieh Jahangard, Ali Javinani, Amir Ashraf-Ganjouei, Parviz Toossi

**Affiliations:** ^1^Skin Research Center, Shahid Beheshti University of Medical Sciences, Tehran, Iran; ^2^Rheumatology Research Center, Tehran University of Medical Sciences, Tehran, Iran

## Abstract

**Background:**

Morphea is an inflammatory disease of the connective tissue that may lead to thickening and hardening of the skin due to fibrosis. The aim of this study was to document magnetic resonance imaging (MRI) changes in patients with linear morphea who were treated with methotrexate (MTX) and high-dose corticosteroid.

**Methods:**

This study was conducted on 33 patients from the outpatient's dermatology clinic of our institute, who fulfilled the inclusion criteria. Patients received 15 mg/week of MTX and monthly pulses of methylprednisolone for three days in six months. The effectiveness of the treatment was evaluated by MRI, modified LS skin severity index (mLoSSI), and localized scleroderma damage index (LoSDI).

**Results:**

All parameters of mLoSSI and LoSDI including erythema, skin thickness, new lesion/lesion extension, dermal atrophy, subcutaneous atrophy, and dyspigmentation were also noticeably improved after treatment. Subcutaneous fat enhancement was the most common finding in MRI. MRI scores were significantly associated with clinical markers both before and after the treatment with the exception of skin thickness and new lesion/lesion extension which were not associated with MRI scores before and after the treatment, respectively.

**Limitations:**

The lack of correlative laboratory disease activity markers, control group, and clearly defined criteria to judge the MRI changes.

**Conclusion:**

MRI could be a promising tool for the assessment of musculoskeletal and dermal involvement and also monitoring treatment response in patients with morphea.

## 1. Introduction

Morphea, localized scleroderma (LS), is an inflammatory connective tissue disease that may lead to skin thickening and hardening due to fibrosis. The course of LS includes an early inflammatory stage. It starts with hyperaemia of the skin and is followed by fibrosis, sclerosis, and, finally, atrophy [1]. LS has a diverse clinical presentation [2] and has been classified into circumscribed (superficial and deep), linear (including scleroderma en coup de sabre), generalized (four or more and larger than 3 cm individual plaques), pansclerotic, and mixed forms [3].

Morphea affects the underlying structures [3], in which case it may be associated with flexion contractures, pain, and considerable impairment [4, 5]. At the more severe end of the spectrum, the disease can progress over the years and cause significant atrophy, joint contractures, irreversible structural deformities, growth retardation, and severe functional, cosmetic, and psychological disabilities [6].

Although numerous therapeutic modalities including methotrexate (MTX) in combination with systemic corticosteroid have been discussed for these debilitating forms of morphea, optimal treatment is unknown maybe due to a lack of consensus as to the methods of evaluation and monitoring of treatment response [7–13].

Although several clinical and paraclinical diagnostic and assessment tools including the modified LS skin severity index (mLoSSI), the localized scleroderma cutaneous assessment tool (LoSCAT), ultrasonography (US), elastography, and skin biopsy have been documented, all of them have some limitations; for example, only a limited subset of the clinical parameters is evaluated by each mLoSSI and LoSCAT, or elastography is so hard to perform, US is usually not so helpful on the scalp/face due to a thin tissue, and finally no one really wants to biopsy the face. Magnetic Resonance Imaging (MRI) has recently been shown to be a useful diagnostic tool for identifying the musculoskeletal involvement in patients with morphea [14–16].

The aim of this study was to document the MRI changes in patients with morphea who were treated with MTX and high-dose corticosteroid.

## 2. Materials and Methods

### 2.1. Study Population

The study was approved by the ethics committee of Shahid Beheshti University of Medical Sciences. Informed consent was sought from the patients in accordance with legal requirements.

This study was conducted on 33 patients from the outpatient's dermatology clinic, who fulfilled the inclusion criteria. The inclusion criteria were immunocompetent patients 10 years old or older with confirmed morphea (generalized or linear forms), normal bone mineral density, and being negative for latent infections, endocrinopathies, and cardiovascular, renal, and pulmonary disorders. The patients were excluded if they had systemic sclerosis, major concomitant medical conditions, leukopenia <3.0 × 10^9^/l, thrombocytopenia <100 × 10^9^/l, liver transaminase levels more than twice the upper limit of normal, or renal impairment defined as creatinine clearance <90 ml/min/1.73 m^2^, osteopenia, and osteoporosis, or if the patient was unwilling or unable to adhere to the protocol.

Morphea diagnosis was confirmed by histological examination. Owing to the activation of latent infections during immunosuppressive treatment regimens, the patients were also examined for tuberculosis, viral hepatitis, and HIV infections.

### 2.2. Treatment Protocol

Treatment protocol and duration encompassed six months and, in this period, the patients received MTX, methylprednisolone, and folic acid. MTX was started with a weekly oral dosage of 15 mg, and 1 mg of daily folic acid was prescribed to diminish its toxicity. At the beginning of each month, patients were hospitalized for three days to administer 20–30 mg/kg (500–1000 mg/m^2^) per pulse, up to a maximum dose of 1 g intravenous methylprednisolone (or 2 mg/kg/day of prednisolone with a maximum dose of 60 mg and 1 mg/kg/week of MTX with a maximum dose of 15 mg/week in pediatrics). On the other hand, all patients were followed up to 18 months and methotrexate (15 mg/wk) was continued in this period.

### 2.3. Clinical Assessment

The localized scleroderma assessment tool (LoSCAT) assesses 18 cutaneous anatomic sites, capturing both disease activity and damage parameters. Scores for each site are based on the most severe score for each parameter. To minimize intersubject variability, all skin changes are compared with the contralateral or ipsilateral skin area.

The modified LS skin severity index (mLoSSI) includes the total of three separate activity scores as follows: (A) erythema (ER), (B) skin thickness (ST), and (C) new lesion/lesion extension (N/E). Three cutaneous damage domains are summated to obtain the localized scleroderma damage index (LoSDI) as follows: (A) dermal atrophy (DAT), (B) subcutaneous atrophy (SAT), and (C) dyspigmentation (DP) [17].

Patients were visited monthly with complete laboratory data to check for probable adverse side-effects. Efficacy of the treatment was assessed clinically by mLoSSI and LoSDI before starting the medication. MRI was also performed before and after 6 months of the treatment by 1.5T closed-bore MRI body scanner which elucidates the depth and thickness of the soft-tissue structures and the degree of inflammation and edema. MRI scans were performed for all patients using high-performance gradients (maximum amplitude, 45 mT/m; minimum rise time, 200 *μ*s; maximum slew rate, 200 T/m/s) with integrated parallel acquisition techniques in three spatial directions. The imaging protocol consisted of gradient-echo localizers on each table position, followed by coronal whole-body STIR images for assessment of pathological signal changes occurring in the musculoskeletal system. MRI images were graded from 0 to 10 by an experienced and blinded radiologist. Also MRI scores (before and after treatment) were compared and scored from 0 (no improvement) to 10 (total healing) by investigators.

### 2.4. Statistical Methods

The variables were checked for having normal distribution by using Shapiro-Wilk test. According to nonparametric clinical variables (LoSDI, mLoSSI, and MRI), the Wilcoxon signed-rank test was used instead of paired-samples* t*-test. The correlation of these clinical markers with MRI scores before and after treatment was also assessed separately using Spearman's correlation test. Variations in clinical indices were analyzed by two-tailed Student's* t*-test with odds ratio (OR) and 95% confidence interval (CI). P-values which were adjusted with Benjamini–Hochberg method to control the false discovery rate lower than 0.05 were considered statistically significant. SPSS software for Windows (version 19.0, IBM SPSS Inc., USA) was used to perform all statistical analyses.

## 3. Results

### 3.1. Demographic Features

In present study, the female to male ratio was 5.6:1 (28 women and five men). The median age was 29.00 with IQR_25-75_ of 19.00 to 37.50, with an age range of 10–61 years. Eight patients (24.2%) were <18 years. The mean duration of skin stiffness or atrophy was 5.81 ± 3.31 months, and the minimum and maximum durations were two and 13 months, respectively. Linear morphea was diagnosed in 28 cases that all of them had the en coup de sabre subtype; none of these patients had neurological involvement. The remaining five individuals were diagnosed with generalized type.

### 3.2. Efficacy of Treatment

As mentioned previously, the efficacy of the treatment was evaluated by mLoSSI, LoSDI, and MRI, which is shown in Table 1. Based on statistically acceptable number of linear morpheae, the efficacy of the treatment has been evaluated separately in this group of patients and shown in Table 1. The number of cases with generalized morphea was negligible to analyze them distinctly.

**Figure 1:**
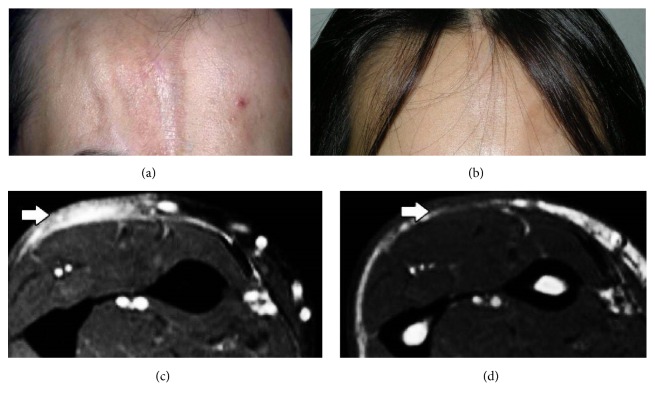
A patient with linear morphea before (a) and after (b) treatment. MRI results before (c) and after (d) treatment.

According to mLoSSI, erythema, skin thickness, and new lesion/lesion extension were significantly improved after the treatment. Furthermore, all parameters of LoSDI including dermal atrophy, subcutaneous atrophy, and dyspigmentation were also noticeably improved after treatment. The treatment regimen was evaluated to be an effective method according to MRI by a P-value <0.001 (Figure 1). The results identified subcutaneous fat enhancement as the most common finding. The association of MRI scores with clinical markers (erythema, skin thickness, new lesion/lesion extension, dermal atrophy, subcutaneous atrophy, and dyspigmentation) before and after treatment is shown separately in Table 2. As it is evident, all of these clinical markers have an accepted and significant correlation with MRI modality, either before and after the treatment. The two exceptions were skin thickness and new lesion/lesion extension which were not associated with MRI scores before and after the treatment, respectively.

### 3.3. Safety and Tolerability

Among the 33 patients who received treatment, all of them experienced some adverse side-effects. According to the precise inclusion criteria, which excluded patients with latent infection, none of them experienced any severe adverse effect of immunosuppression.

Weight gain and acne vulgaris were the most prevalent side-effects, detected in 26 and 23 patients, respectively. These were primarily due to high dosages of glucocorticoid. Constitutional side-effects such as fatigue (n=13), nausea (n=11), and headache (n=5) were also reported in less than half of the patients, which could probably be due to MTX. Striae rubrae and alopecia were two cosmetically important adverse effects, respectively, observed in seven and six patients. Secondary Cushing syndrome was observed in approximately 10% of the patients (three individuals) as the most important adverse effect in this study. Finally, hypokalemia (n=8), leukopenia (n=2), and anorexia (n=1) were other reported side-effects.

## 4. Discussion

It is worth noting that this study demonstrated the effectiveness and sensitivity of MRI to treatment and its association with LoSCAT that would definitely be more novel and unique in the treatment of these patients.

Over the years, a variety of therapeutic options has been reported for morphea, topical and systemic corticosteroids, topical and systemic calcipotriol, topical tacrolimus, phototherapy, and MTX [12, 18–23]. One of the most challenging problems in the treatment of patients with morphea is the assessment of treatment efficacy and duration of the treatment. A valuable follow-up instrument should be able to differentiate between therapy responders, patients with stable disease, and those who are unresponsive to treatment. Mertens et al. showed that recurrences in morphea can occur even after many years of quiescence [24].

In the study conducted by Kreuter et al., fourteen LS patients were treated with 15 mg/week of MTX and monthly methylprednisolone pulse (1000 mg for three days) for six months. The effectiveness of the treatment was evaluated by ultrasound (US) and histological findings. Almost all of the patients had achieved clinical improvement, which was confirmed by paraclinical criteria [11]. In another study, Torok et al. examined the effectiveness of 1 mg/kg/week of MTX (a maximum dose of 25 mg/week) and 2 mg/kg/day of prednisone (a maximum dose of 60 mg). This treatment regimen was prescribed for 36 pediatrics patients who were followed up for an average of 36 months. The effectiveness was measured by physician general assessment (PGA) and mLoSSI, which showed clinical improvement in all patients [25]. Another study performed by Seyger et al. analyzed the effectiveness of 15 mg/week of MTX using visual analogue scale (VAS), durometer score, and modified skin score (MSS). After 36 weeks of follow-up, improvement in all patients was observed [26]. A multicenter study was published in 2006, in which methylprednisolone pulse (a maximum dosage of 500 mg) and 10 mg /m^2^/week of MTX were given to 34 patients. The effectiveness of the treatment was measured by thermography and clinical criteria, and, ultimately, 71% of patients had entered the remission phase [6].

Several clinical and laboratory methods have been reported to measure morphea disease activity. One of these criteria is LoSCAT. Arkachaisri et al. [27] in 2008 examined the reliability of these criteria. In this study, two rheumatologists registered these symptoms for 22 patients and reported a significant association of LoSSI with medical interventions. Also they showed a significant association of mLoSSI with PGA, patients' quality of life, and medical interventions over time [28].

In 2010, a new standard was designed for the measurement of damage that was composed of DT, SAT, and DP. The reliability of this score with mLoSSL and PGA was acceptable. In our study, mLoSSI and LoSDI were significantly improved after treatment. Similar to earlier studies [25], we also used LoSCAT which was significantly improved. But as mentioned earlier, each evaluates only a limited subset of the clinical parameters especially that they are not so sensitive in the en coup de sabre subtype.

An important issue is the lack of a universal agreement on the optimal tools to be used for assessment of disease activity and damage severity or monitoring of the treatment response. Skin scores and recently more sensitive tools including laser Doppler flowmetry, Doppler US, and MRI have been used for evaluation and monitoring of the lesions [29].

Ultrasonography has been utilized for assessment of the superficial linear and circumscribed lesions because of its availability and excellent soft-tissue resolution, but evaluation of deep tissues may be impossible by US [14] and also US is usually not helpful as much as MRI on the scalp/face due to a thin tissue.

The data clearly show that MRI plays a key role and is able to document both dermal and musculoskeletal involvement at the initial presentation, as well as the changes occurring during these therapies. A valuable follow-up instrument should be able to differentiate between therapy responders, patients with stable disease, and those who are unresponsive to treatment. In our series, we found treatment success, which is in line with previous studies. Schanz et al. [15] showed that, in patients with morphea, MRI is a useful tool for the detection of clinical manifestations. Consistent with findings of Schanz et al. [16], our results showed that MRI is a suitable tool for patients with morphea and also the musculoskeletal and dermal manifestations in MRI improved in parallel with clinical improvement after systemic treatment.

Schanz et al. [16] mentioned that MRI provided complementary information about the depth of involvement of underlying morphological structures, contrary to clinical examination, which generally reveals information about the superficial involvement in this disorder. Thus, MRI may confirm the findings from the physical examination, and its findings are generally easier to interpret. It is better to evaluate the depth of involvement in generalized or deep morphea (also its subtypes) by imaging techniques (particularly MRI). The signal intensity is based on the grade and level of involvement, but there are no clearly defined criteria to judge the MRI changes [15, 16].

This study is subject to a number of important limitations: the lack of correlative laboratory disease activity markers and clearly defined criteria to judge the MRI changes; lack of a control group can be mentioned as another limitation, but it should be noted that it is inconsistent with ethical principles for patients not treated for several months.

## 5. Conclusions

We demonstrate the ability of MRI to capture disease improvement in patients with linear morphea responding to a methotrexate and corticosteroid regimen as defined by a significant change in the mLoSSI and LoSDI scores. Larger and comparative studies are needed to elucidate the diagnostic and monitoring applications of MRI in different types of morphea.

## Figures and Tables

**Table 1:** Efficacy of the treatment regimen.

**Clinical or Paraclinical Indices**		**Shapiro-Wilk Test**	**Before treatment** Median (IQR_25-75_)	**After treatment** Median (IQR_25-75_)	**P-value** ^*∗*^
**mLoSSI**	Erythema	<0.001	2.00 (2.00-2.00)	1.00 (1.00-1.50)	<0.001
	Skin thickness	<0.001	1.00 (1.00-2.00)	1.00 (1.00-1.50)	0.002
	New lesion/lesion extension	<0.001	1.00 (1.00-1.50)	0.00 (0.00-0.00)	<0.001

**LoSDI**	Dermal atrophy	<0.001	2.00 (2.00-2.00)	1.00 (1.00-1.00)	<0.001
	Subcutaneous atrophy	<0.001	1.00 (0.00-2.00)	1.00 (0.00-2.00)	0.014
	Dyspigmentation	<0.001	2.00 (2.00-3.00)	1.00 (1.00-2.00)	<0.001

**Magnetic Resonance Imaging**		0.004	4.00 (3.00-6.00)	2.00 (1.00-3.00)	<0.001

LoSDI: localized scleroderma damage index; mLoSSI: modified localized skin severity index; *∗* the Wilcoxon signed-rank test was used and adjusted P-value is reported according to Benjamini and Hochberg test.

**Table 2:** The association between MRI and mLoSSI and LoSDI.

**Variables**	**Before treatment** ^**∗**^	**P-value** ^**ǂ**^	**After treatment** ^**∗**^	**P-value** ^**ǂ**^
**Erythema**	0.479	0.008	0.467	0.008

**Skin thickness**	0.345	0.053	0.466	0.008

**New lesion/lesion extension**	0.451	0.009	0.322	0.068

**Dermal atrophy**	0.528	0.004	0.558	0.002

**Subcutaneous atrophy**	0.642	<0.001	0.585	<0.001

**Dyspigmentation**	0.536	0.002	0.596	<0.001

*∗* correlation coefficient with MRI scores is shown in these columns. *ǂ* P-values are adjusted by Benjamini and Hochberg method.

## Data Availability

There are no linked research data sets for this article. Data will be made available on request

## Abbreviations

LS: Localized scleroderma
MTX: Methotrexate
MRI: Magnetic resonance imaging
mLoSSI: Modified LS skin severity index
LoSDI: Localized scleroderma damage index.
